# Differences in the genotype frequencies of genes related to blood pressure regulation - a comparative study between South-West Europe and Peri-equatorial Africa

**DOI:** 10.4314/ahs.v21i4.21

**Published:** 2021-12

**Authors:** Laura Aguiar, Ildegário Semente, Joana Ferreira, Andreia Carvalho, Alda P Silva, Cristina Caroça, Helena Caria, Albertino Damasceno, Maria J Laires, Luís Sardinha, Cristina Monteiro, Mário R Mascarenhas, Paula Faustino, Ângela Inácio, Manuel Bicho

**Affiliations:** 1 Instituto de Investigação Científica Bento da Rocha Cabral, Lisboa, Portugal; 2 Laboratório de Genética, Faculdade de Medicina da Universidade de Lisboa, Lisboa, Portugal; 3 Instituto de Saúde Ambiental, Faculdade de Medicina da Universidade de Lisboa, Lisboa, Portugal; 4 Departamento de Genética Humana, Instituto Nacional de Saúde Doutor Ricardo Jorge, Lisboa, Portugal; 5 Departamento de Otorrinolaringologia, Faculdade de Ciências Médicas, Universidade Nova de Lisboa, Portugal; 6 Hospital CUF Infante Santo, Lisboa, Portugal; 7 BioISI - Biosystems & Integrative Sciences Institute, Faculty of Sciences, University of Lisbon, Portugal; 8 Departamento de Ciências Biomédicas, Escola Superior de Saúde, Instituto Politécnico de Setúbal, Setúbal; 9 Faculdade de Medicina da Universidade Eduardo Mondlane, Maputo, Moçambique; 10 Hospital Central de Maputo, Maputo, Moçambique; 11 Centro de Estudos da Performance Humana, Faculdade de Motricidade Humana da Universidade de Lisboa, Lisboa, Portugal; 12 Clínica de Endocrinologia, Diabetes e Metabolismo de Lisboa, Lisboa, Portugal

**Keywords:** Blood pressure, genetics, Africa

## Abstract

**Background:**

Since the emergence of the genus Homo, hominids have occupied a wide variety of environments, facing different selective pressures.

**Objectives:**

The aim this study is to compare genotype frequencies between South-West Europe and Peri-equatorial Africa in genes potentially modulators of blood pressure.

**Methods:**

The analyzed sample consisted of 325 individuals from Portugal and 226 individuals from Africa (48 from Mozambique and 178 from São Tomé and Príncipe). The following genetic variants were analyzed: intron 4 VNTR in eNOS, rs1050829 in G6PD, -3.7kb α-thalassemic deletion in HBA, rs1800457 in CYB5R3, Hp 1/2 genotype/phenotype in Hp and intron 16 I/D in ACE.

**Results:**

Frequencies of genotypes with the 4a allele in eNOS (p<0.001), the G allele in G6PD (p<0.001), the α-3.7 kb in HBA (p <0.001), the C allele in the CYB5R3 (p<0.001) were higher in Peri-equatorial Africa. The Hp 1.1 genotype of Hp has a higher frequency in Peri-equatorial Africa (p=0.002). ACE shows no significant differences.

**Conclusion:**

Results show differences in five genetic variants. Conditions of extreme heat and humidity, characteristic of Peri-equatorial Africa, have been associated with increased sodium loss. This study suggests that selected compensatory mechanisms printed in the genome, are nowadays risk factors for hypertension in Peri-equatorial Africa.

## Introduction

Blood pressure is controlled by complex mechanisms not yet completely clarified. However, it is well accepted that maintenance of normal blood pressure depends on the interaction between genes and environment and that genetic variability may play an important role in the pathological process of hypertension^1^,^2^. In fact, in recent years several genes have been associated with blood pressure control and susceptibility to hypertension^3^–^6^.

Nitric oxide (NO) is synthesized from L-arginine by endothelial nitric oxide synthase (eNOS) and plays an important role in several physiological processes. It is a potent vasodilator agent allowing the maintenance of vascular tonicity and blood pressure regulation^7^–^9^.

Another enzyme that plays an important role in the pathophysiology of hypertension is glucose-6-phosphate dehydrogenase (G6PD). This enzyme generates nicotinamide adenine dinucleotide phosphate (NADPH), a co-factor for NO synthesis, and its deficiency is associated with decreased NO^10^. Several studies have found an association between G6PD enzyme deficiency and hypertension or/and cardiovascular disease^11^–^13^.

Endothelial alpha-globin (Hb α) and cytochrome b5 reductase 3 (Cyb5R3) may also regulate NO bioavailability. An interaction mechanism has been described between Hb α and eNOS, both forming a macromolecular complex in the myoendothelial junctions, important for NO signaling between endothelium and smooth muscle^14^. Moreover, NO diffusion from endothelium towards smooth muscle is regulated by the oxidative state of the Hb α heme group, with NO having a high affinity to iron in its reduced form^15^–^18^. Cyb5R3 is a methemoglobin reductase that controls the oxidative state of the Hb α heme group by reducing Fe3 + to Fe2+^17^.

One of the most studied markers of sodium susceptibility to blood pressure over the past years is haptoglobin (Hp). Hp is an acute phase plasma glycoprotein characterized by an inter-individual molecular variation, its main function is to bind free hemoglobin (Hb), forming a complex that will be eliminated by monocytes and hepatocytes^19^. As part of the free Hb elimination mechanism, Hp will act as an antioxidant, protecting against free radicals^20^. There are three distinct genotypes / phenotypes (Hp 1.1, Hp 2.1 and Hp 2.2) that bind to Hb with different affinities^21^.

Angiotensin converting enzyme I (ACE) is an enzyme that plays a key role in blood pressure control by catalyzing the conversion of angiotensin I to angiotensin II, a potent vasoconstrictor and a aldosterone production enhancer that increases sodium retention^22^,^23^, thus deviations on its regular function may be implicated in pathogenesis of high blood pressure.

Following the emergence of the genus Homo, hominids occupied a wide variety of environments with different temperatures and humidity, as a result, selective pressures are expected to vary between geographically distinct regions^24^,^25^. Several studies report an increased susceptibility to hypertension in populations ancestry from peri-equatorial environments, in association with an adaptive cardiovascular regulation of salt loss by perspiration^26^–^28^.

The aim of the present work is to compare genotype frequencies in genes potentially modulators of blood pressure, in South-West Europe and Peri-equatorial Africa. Genes are endothelial nitric oxide synthase (eNOS), glucose-6-phosphate dehydrogenase (G6PD), alpha globin (HBA), cytochrome b5 reductase 3 (CYB5R3), haptoglobin (Hp) and angiotensin converting enzyme I (ACE).

## Methods

### Subjects

In this work we studied a group of 325 individuals from South-West Europe (Portugal), and 226 from Peri-equatorial Africa (48 from Mozambique plus 178 from São Tomé and Principe). The study protocol was approved by the local Ethical Committee. Written informed consent was obtained from all participants and the study was conducted in line with the principles of the Declaration of Helsinki. Because, sometimes we could not obtain high quality data and it was not possible to repeat sample collection, there is a different N number per gene.

### Genomic DNA Isolation

Whole blood samples from patients and controls were stored with EDTA at −20°C. The genomic DNA was isolated through a non-enzymatic method adapted from Lahiri and Numberger (1991)29.

### eNOS Genotyping

Analysis of the variable number tandem repeats (VNTR 27 bp-4a/b) in intron 4 of the eNOS gene, was made by PCR screening. PCR was carried out in a 25 µL reaction volume, containing ≈ 200 ng of genomic DNA, 1 µL (20 pmol) of each of sense and antisense primers: 5′AGGCCCTATGGTAGTGCCTTT3′ and 5′TCTCTTAGTGCTGTGGTCAC3′. These primers amplify a fragment with 293 bp (4a) or 420 bp (4b). The PCR program included a step of 94 °C, for 5 min followed by 30 cycles of denaturation at 94 °C for 30 s, annealing at 55 °C for 45 s and extension at 72 °C for 30 s. An additional extension step was performed in the final at 72 °C for 5 min. PCR products were separated on 3% agarose gel with ethidium bromate staining and visualized under UV light.

### G6PD Genotyping

The rs1050829 of the G6PD gene was characterized by PCR followed by Restriction Fragment Length Polymorphism (RFLP). PCR was carried out in a 25 µL reaction volume, containing ≈ 200 ng of genomic DNA, 1 µL (20 pmol) of each of sense and antisense primers: 5′CTGCGTTTTCTCCGCCAATC3′ and 5′AGGCAACGGCAAGCCTTAC3′. These primers amplify a fragment with 585 bp. The PCR program included a step of 94 °C, for 5 min followed by 30 cycles of denaturation at 94 °C for 30 s, annealing at 62,8 °C for 30 s and extension at 72 °C for 30 s. An additional extension step was performed in the final at 72 °C for 5 min. PCR products were separated on 2% agarose gels with ethidium bromate staining and visualized under UV light. The amplified fragment was then restricted by Fok I (enzyme from New England Biolabs, Germany) originating fragments of 402 bp plus 187 bp (higher G6PD activity) or 289 bp, 187 bp plus 117 bp (lower G6PD activity). The results were visualized in a 1% agarose gel ethidium bromate stained and visualized under UV light.

### α-globin Genotyping

For the α-3,7kb thalassemic deletion, a Gap-PCR was performed. PCR was carried out in 25 µL reaction volume, containing ≈ 200 ng of genomic DNA, 1 µL (20 pmol) of sense primers (5′GGGATGCACCCACTGGCACT3′) plus 1 µL (20 pmol) of each antisense primer (5′CTCCATTGTTGGCACATTCCGGG3′ and ´CTGCTGTCCACGCCCATGCC3′). These primers amplify a fragment with 2100 bp (no deletion) or 1900 bp (deletion). The PCR program included a step of 94 °C, for 5 min followed by 30 cycles of denaturation at 94 °C for 60 s, annealing at 64 °C for 60 s and extension at 72 °C for 150 s. An additional extension step was performed in the final at 72 °C for 5 min. PCR products were separated on 1% agarose gel with ethidium bromate staining and visualized under UV light.

### CYB5R3 Genotyping

For the rs1800457 characterization on CYB5R3 gene, a PCR end Point analysis was performed using a MyGo Pro® Real time PCR detection System. The sequences of the labeled probe were CCCTCCAGCGGGAAACTTGGGATGG[C/G] TGTCCTTGAAGTAAACCTGCAAGAC (VIC/FAM) (Assay ID C___2986212_20, Thermo Fisher Scientific, Portugal). Thermal cycling conditions consisted of an initial denaturation step at 95 °C for 10 min, 40 cycles of 95 °C for 15 s, and 60 °C for 60 s. PCR data were analyzed using MyGo Pro PCR Software 3.4.

### Haptoglobin Genotyping

The Hp genotype was determined by polyacrylamide gel electrophoresis (PAGE). A 10% hemoglobin solution in water was prepared from heparinized blood by first washing the blood cells five times in phosphatebuffered saline (0.1 mol/L, pH 7.2) and then lysing the cells in 9 mL of sterile water per milliliter of pellet cell volume. A supernatant of a cell lysate containing hemoglobin was aliquoted in 1 or 0.5 mL and stored at -20C. Hp phenotyping was determined by gel electrophoresis and peroxidase staining, using a modified version of the method described previously30. Briefly, serum (20mL) was mixed with 10 mL of the 10% hemoglobin solution and 15 mL of 40% saccharose, and the samples were left to stand for 5 minutes at room temperature to allow the formation of Hp-Hb complexes. The Hp-Hb complex was resolved by PAGE using a buffer containing 50 mmol/L Tris base and 384 mmol/L glycine. The gel was 14 mL of 40% acrylamide/bis-acrylamide in 14 mL of 3 mol//L Tris-HCl, pH 8.9 and 21 mL of bidestilated water. Three hundred and fifty microliters of N,N,N′,N′-Tetramethylethylenediamine and 1 mL of ammonium persulphate (12 mg/mL) were added to the previous solution. After the completion of electrophoresis, which was performed at a constant voltage of 250 for 4 hours, the Hp-Hb complexes were visualized by soaking the gel in two freshly prepared staining solutions in a glass tray. The first staining solution contained ortho-dianisidine 5 mg/mL in 50% (vol/vol) glacial acetic acid, and the second one was made of 2% (vol/vol) hydrogen peroxide. The bands corresponding to the Hp-Hb complex were readily visible within 15 minutes and were stable for more than 48 hours.

### ACE Genotyping

The analysis of the I/D (insertion/deletion) on the ACE gene was screening in DNA samples by a PCR approach. PCR was carried out in a 25 µL reaction volume, containing ≈ 200 ng of genomic DNA, 1 µL (20 pmol) of each of sense and antisense primers: 5′GCCCTGCAGGTGTCTGCAGCATGT3′ and 5′GGATGGCTCTCCCCGCCTTCTCTC3′. These primers amplify a fragment with 319 bp (D) or 597 bp (I). The PCR program included a step of 94 °C, for 5 min followed by 30 cycles of denaturation at 94 °C for 60 s, annealing at 58 °C for 60 s and extension at 72 °C for 60 s. An additional extension step was performed in the final at 72 °C for 5 min. PCR products were separated on 2% agarose gel with ethidium bromate staining and visualized under UV light.

### Statistical analysis

All tests were performed with SPSS 24.0 software. Differences between the two groups (South-West Europe and Peri-equatorial Africa) were tested with Pearson's chi-squared test or Fisher's Exact test when more than 20% of the data had less than 5 counts. Statistical significance was defined as a p-value < 0.05.

## Results

Comparison of the genotype frequencies between Peri-equatorial Africa and South-West Europe in eNOS, G6PD, HBA, CYB5R3, Hp and ACE genes is shown in Table 1. It is possible to observe that, apart from ACE gene, after clustering some genotypes, distributions are different between the two geographical localizations. Regarding eNOS, genotypes with the 4a allele (4a/4a and 4a/4b) have a higher frequency in Peri-equatorial Africa (64,6 %) than in South-West Europe (32,5 %) (p<0,001). For G6PD, there is a higher frequency of genotypes with the G allele (G, GG and AG) in Peri-equatorial Africa (45,7 %) than in South-West Europe (2,1 %) (p<0,001). The presence of the α-3,7kb deletion in the HBA gene (α-3,7/α-3,7 and αα/α-3,7) is common in Africa (72,7 %) but rare in South-West Europe (1,2 %) (p<0,001). Concerning CYB5R3, there is a higher frequency of genotypes carrying the C allele (CC and GC) in Peri-equatorial Africa (50,0 %) than in South-West Europe (5,9 %) (p<0,001). In relation to HP, there is a higher frequency of genotypes Hp 1.1 in Peri-equatorial Africa (37,5%) once compared with South-West Europe (16,8%) (p=0,002). ACE shows no significant differences in genotype distribution between the two geographical regions.

**Table 1:** Comparison of genotype frequencies between Peri-equatorial Africa and South-West Europe - *eNOS, G6PD, HBA, CYB5R3, Hp* and *ACE* genes

Gene (variant)	Genotype	Peri- equatorial Africa N (%)	South-West Europe N (%)	P value
*eNOS* (VNTR intron 4)	4a/4a and 4a/4b	51 (64,6)	51 (32,5)	<0,001^1^
	4b/4b	28 (35,4)	106 (67,5)	

*G6PD* (rs1050829)	G, GG and AG	91 (45,7)	3 (2,1)	<0,001^1^
	A and AA	108 (54,3)	137 (97,9)	

*HBA* (α /α-3,7kb)	α-3,7/α-3,7 and αα/α-3,7	16 (72,7)	1 (1,2)	<0,001^2^
	αα/αα	6 (27,3)	81 (98,8)	

*CYB5R3* (rs1800457)	CC and GC	59 (50,0)	3 (5,9)	<0,001^1^
	GG	59 (50,0)	48 (94,1)	

*Hp* (protein phenotype 2.2/2.1/1.1)	2.2 and 2.1	30 (62,5)	154 (83,2)	0,002^1^
	1.1	18 (37,5)	31 (16,8)	

*ACE* (Insertion/Deletion)	DD and ID	39 (92,9)	213 (89,5)	0,503^2^
	II	3 (7,1)	25 (10,5)	

1 Pearson's χ2 test – n (%)

2 Fisher's Exact test – n (%)

## Discussion

The epidemics of hypertension is certainly related with nowadays environmental risk factors, such as excessive salt consumption or eating behaviors leading to obesity. However, also genetic susceptibility contributes to this disease onset. Different populations present different susceptibilities, with populations originated from hot and humid environments, being more susceptible than populations from cold environments^26^–^28^. This is probably due, not only to cultural (consanguinity) and infectious factors^31^ (positive selection in G6PD and HBA by malaria), but also to a historic adaptation of the human being to climate^27^. Indeed, factors that nowadays increase susceptibility to hypertension, such as vascular reactivity and sodium and water retention, may have been adaptive in an ancestral African environment characterized by a hot, humid and salt-poor climate^28^,^32^.

In this study there are significant differences in eNOS genotype frequencies between the two groups, with the smaller allele (4a - four VNTR of 27 bp) in intron 4 (a/b) being more frequent in Africa. This result is in accordance with the literature, that reports differences between African and European populations^33^. The 4a allele is associated with a lower gene expression leading to a reduction in the NO levels and consequently to a loss of endothelium integrity 34. In fact, some studies found an association between the 4a allele and the susceptibility to hypertension and cardiovascular risk^35^–^38^.

Concerning rs1050829 in the G6PD, in accordance with other reported results^39^, there is a higher frequency of the G containing genotypes in Africa. This variant is associated with lower levels of the enzyme^40^,^41^, providing lower amounts of NADPH to act as cofactors for the NO production and potentially leading to vascular disfunction and hypertension.

Regarding HBA gene, it was found an association of the -α3,7Kb variant with Africa. Other studies show the same^42^–^44^. This deletion may disturb the Hb α/eNOS interaction and compromise the macromolecular complex stability in myoendothelial joints, interfering with the regular NO signaling between endothelium and smooth muscle. Likewise, for the CYB5R3 gene, results are in accordance with other studies, showing the C containing allele being more frequent in Africa^45^. Results obtained from our group revealed an association between rs1800457 and a higher activity of the enzyme in Sickle Cell Disease patients (not published). Other authors showed an association between high Cyb5R3 levels and hypertension^46^.

In accordance with previous studies, we also detected a higher frequency of the Hp 1.1 genotype in Africa^47^. Other authors have associated the Hp 2.2 genotype with susceptibility of blood pressure to sodium^46^,^48^–^50^. One possible explanation is the lower protection that Hp 2 provides against vascular complications, since it binds with a lower affinity to Hemoglobin^51^. On the other hand, Hp 1 is a potent inhibitor of PGE2 biosynthesis, a natriuretic product^52^. Schaer et al. demonstrated that Hemoglobin binding by Hp restores vascular NO signaling during hemolysis^53^ consequently, for the higher frequency of the Hp 1.1 genotype in Africa may also contributed a selection of the Hp 1 allele in a context of highly frequent hemolytic diseases.

Several studies have demonstrated an association between hypertension and ACE gene. One of the most studied polymorphism in this gene is the I/D, characterized by an insertion/deletion of 287 bp in intron 6, being the D allele associated with physiologic alterations that can lead to hypertension development^54^,^55^. Previous studies that compared the I/D distribution between Caucasians and sub-Saharan Africans descendants, show a higher frequency of the D allele in the second ones^56^,^57^. However, our results, in agreement with Sagnella et al. do not find significant differences between South-West Europe and Peri-equatorial Africa^58^.

Effective heat dissipation is essential in hot environments and is most efficiently achieved through perspiration, which can lead to loss of large amounts of salt and water^59^. Under these conditions, salt desire and renal sodium conservation are essentials for survival therefore, in the past, humans and nonhuman primates from tropical climates increased their salt avidity^60^–^63^. Perspiration also causes a drop in blood volume^64^, under these conditions, compensatory mechanisms involved in increasing arterial tone and cardiac contraction force, maintain regular blood pressure values. Thus, genetic variation that increases arterial and cardiac contractility may have conferred survival advantage in the environmental context of early human evolution in Peri-equatorial Africa. Today, after civilization, Africans are less exposed to extreme environmental conditions, highlighting the adaptive compensatory mechanisms acquired over time by selection, in the form of hypertension. However, other selective pressures such as malaria (positive selection for G6PD polymorphism and α-3.7kb) and cultural habits such as inbreeding, surely also contributed for the selection of risk genotypes for hypertension.

## Conclusion

In the present study we found differences in the genotypic distributions of five genetic variants between two distinct geographic regions - South-West Europe and Peri-equatorial Africa, that are involved in the cardiovascular regulation and potentially influence the development of hypertension. To this contrast may have contributed, different selective pressures associated with the distinct climates. In fact, conditions of extreme heat and humidity are associated with increased sodium loss, which is relevant to blood pressure maintenance. Thus, this study suggests that initially selected compensatory mechanisms that become printed in the genome, are now risk factors for the development of hypertension.
